# Is robotic lobectomy cheaper? A micro-cost analysis

**DOI:** 10.1007/s11701-022-01377-x

**Published:** 2022-02-28

**Authors:** Ben Shanahan, Usha S. Kreaden, Jan Sorensen, Steven Stamenkovic, Karen C. Redmond

**Affiliations:** 1 Professor Eoin O’Malley National Thoracic and Transplant Centre, Mater Misericordiae University Hospital, Eccles St., Dublin, D07R2WY Ireland; 2 School of Postgraduate Studies, Royal College of Surgeons in Ireland, Dublin, Ireland; 3 Fellow, Biostatistics & Global Access Value & Economics, Intuitive Surgical Inc., Sunnyvale, CA USA; 4 Healthcare Outcomes Research Centre, RCSI University of Medicine and Health Sciences, Dublin, Ireland; 5 The Thorax Centre, Barts Health NHS Trust, London, UK; 6 School of Medicine and Medical Science, University College Dublin, Dublin, Ireland

**Keywords:** Thoracic surgery, Robotic surgery, Cost analysis, Lobectomy, Healthcare economics

## Abstract

Higher capital costs and operating room costs associated with Lobectomy via Robot Assisted Thoracic Surgery (RATS) have previously been suggested as the principal contributors to the elevated overall cost. This study uses a micro-costing approach to a previous analysis of clinical outcomes of RATS, Video-Assisted Thoracic Surgery (VATS) and Open Lobectomy to evaluate the most significant cost drivers for the higher cost of robot-assisted lobectomy. A micro-costing model was developed to reflect the pathway of patients from day of surgery through the first 30 days following lobectomy. Costs were provided for RATS, VATS and Open approaches. Sensitivity analysis was performed specifically in the area of staff costs. A threshold sensitivity analysis of the overall cost components was also performed. Total cost per case for the RATS approach was €13,321 for the VATS approach €11,567, and for the Open approach €12,582. The overall cost differences were driven primarily by the elevated consumable costs associated with RATS Lobectomy. Capital costs account for a relatively small proportion of the per-case cost difference. This study presents a detailed analysis of the cost drivers for lobectomy, modelled for the three primary surgical approaches. We believe this is a useful tool for surgeons, hospital management, and service commissioning agencies to accurately and comprehensively determine where cost savings can be applied in their programme to improve the cost-effectiveness of RATS lobectomy.

**Supplementary Information:**

The online version contains supplementary material available at 10.1007/s11701-022-01377-x.

## Introduction

Lung cancer remains the leading cause of cancer death in men and women around the world [1]. Surgical resection remains the treatment of choice for early stage Non-Small Cell Lung Cancer (NSCLC), offering the best long-term survival to patients when compared to radiotherapy and other treatments [2]. The advent of Video-Assisted Thoracoscopic Surgery (VATS) in the late 1990s heralded major change for lung cancer patients. The superiority of VATS over open surgery in terms of reduced Length of Stay (LOS) and complication rates has been demonstrated [3, 4]. It is also now widely recognised that long-term survival and oncologic outcomes are similar with the two approaches [5, 6].

In tandem with the rise of VATS in thoracic surgery, robotic surgery was gaining popularity in other specialties. Although originally designed with cardiac surgery in mind, more favourable results in the fields of urology, general surgery and gynaecology accelerated its use in these specialties [7]. Robotic-Assisted Thoracic Surgery (RATS), followed on swiftly, with the first robotic lobectomy for primary lung cancer reported in 2002 [8].

In a recently published meta-analysis, our group has demonstrated robotic lobectomy to be a reasonable alternative to VATS and open surgery, superior to open with respect to complications and length of stay, and superior to both VATS and open with respect to 30-day mortality. This current analysis is a follow-on from that publication, updating and extending the literature review to support a micro-cost analysis. This analysis can inform further health economic discussions around commissioning specialist thoracic robotic services in Ireland and the UK [9].

Cost continues to be the principal limiting factor in the adoption of RATS lobectomy by healthcare providers. NHS England, in a clinical commissioning policy published in 2016, cite capital costs, the learning curve for the team, and lack of tactile feedback as the disadvantages of RATS, and on the basis of this they do not recommend routinely commissioning robotic lung resection for primary lung cancer [10]. Existing cost analyses of RATS lobectomy are derived primarily from early experiences, and reported costs vary greatly. Data from high-volume centres suggest that economies of scale can reduce the cost of robotic lobectomy significantly, with one author estimating that indirect costs, including amortisation of capital cost, maintenance and depreciation account for $1200 of an overall $17,000 cost per case [11]. It is a long-held assumption that the capital cost of robotic equipment is the principal factor in the higher per-case cost of robotic surgery—however, there has been relatively little work done to establish if this is actually the case. Previous analyses of the cost of RATS lobectomy were summarised by Singer and colleagues in 2019. They note that the six retrospective studies considered were limited by variation in cost definitions and by methodological heterogeneity. Interestingly they found that operating room cost was a significant contributor to overall cost differences, more so than capital or consumable equipment costs [12]. The current study therefore set out to provide an in-depth analysis of the cost drivers for RATS, VATS and open lobectomy, with a focus on the perioperative context. Based on this evidence, recommendations regarding optimal usage scenarios for a robotic surgery programme can be considered.

The aim of the current study is to explore the cost of RATS lobectomy with VATS lobectomy and open lobectomy. Cost was evaluated from a 30-day post-operative time perspective, and from an Irish hospital payer perspective. This analysis assumes that longer-term clinical outcomes outside of the 30-day time horizon (pain, functional ability, return to work etc.) are identical across all three approaches. Although that may not be the case, any differences in longer-terms outcomes, and associated cost differences that might be incurred as a result, were not considered by this analysis.

The market for robotic surgery has thus far been dominated by the daVinci platform developed by Intuitive Surgical Inc. (Sunnyvale, Ca, USA). Although other companies, such as CMR Surgical (Cambridge, United Kingdom) and Asensus Surgical (North Carolina, USA), are beginning to enter the market with competing systems, for the purposes of our analysis, we have focused solely on the daVinci system, as it is the only one currently in widespread clinical practice. Our analysis has used the daVinci Xi platform as the base case [13].

## Methods

### Ethics statement

This study is a cost analysis, there were no patients enrolled or clinical outcomes evaluated, and as such specific Institutional Review Board approval was not sought.

### Overview

A deterministic micro-cost model for lobectomy was developed, modelling costs for each of the three surgical approaches (RATS, VATS and Open). The model was structured to represent a patient’s pathway from admission to hospital, through the operation, and into the post-operative period for the first 30 days after the procedure. It was assumed that patients were potential candidates for any of the three approaches, and that there are no contra-indications for any of the approaches for the patient cohort considered. The primary outcome variable was hospital costs accruing in the first 30 days after the procedure.

The major resources (‘cost drivers’) for each approach (RATS, VATS, Open) were first identified. The resource uses were then quantified, drawing on evidence from the established literature, single centre clinical observation and expert opinion where appropriate. Where expert opinion was used, an expert panel consisting of a thoracic surgeon proficient in all three approaches to lobectomy, a health economist and a biostatistician familiar with the current literature regarding the cost of robotic surgery provided this opinion. Irish unit costs in 2020 euros were then attributed to each resource. The product of the resource use and unit cost was then calculated, and this formed the basis for the cost model.

Data sources used to determine resource uses and unit costs are presented in Tables 1, 2, 3, and 4. Certain resource use data were derived from literature review. Resource use data derived from this review included operative time, length of hospital stay, postoperative complication rate, reoperation rate, readmission rate, conversion to open rate and blood transfusion rate. Methodology for this literature review is presented later in this section.

**Table 1:** Staff time allocations and time periods

Staff–time allocation factor	Preparatory	Knife to skin	Postop	Cleaning
Surgeon	0.5	1	0.5	0
Surgical assistant-NCHD	1	1	1	0
Surgical assistant-ANP	1	1	1	0
Anaesthetist	0.5	0.5	0.2	0
Anaesthetic NCHD	1	1	1	0
Anaesthetic Nurse	1	1	1	0
Theatre Nurse Scrubbed	1	1	1	0
Theatre Nurse circulating	1	1	1	0
Portering staff	1	0	1	0
HCA	0	0	0	1

Modality–time period:	Preparatory	Knife to skin	Postop	Cleaning
RATS	30	247.6	20	15
VATS	20	193.6	20	15
Open	20	190.6	20	15

Data sources:

Staff–time allocation factor: expert opinion

Modality–time period: literature review

Key:

*NCHD* non-consultant hospital doctor (junior doctor/resident), *ANP* advanced nurse practitioner, *HCA* healthcare assistant

**Table 2:** Load factors, staff time and costs by modality

Staff member	Annual salary	Load factor	Hourly cost	RATS time	RATS cost	VATS time	VATS cost	Open time	Open cost
Surgeon	142,121	0.5	162	272.0	736	214.0	577	211.0	569
Surgical assistant-NCHD	63,614	0.65	56	298.0	277	234.0	217	231.0	214
Surgical assistant-ANP	65,853	0.75	50	298.0	248	234.0	195	231.0	192
Anaesthetic consultant	142,121	0.5	162	143.0	386	111.0	299	110.0	295
Anaesthetic NCHD	63,614	0.75	50	298.0	248	234.0	195	231.0	192
Anaesthetic nurse	39,180	0.75	30	298.0	148	234.0	116	231.0	114
Theatre nurse scrubbed	41,038	0.75	31	298.0	155	234.0	121	231.0	120
Theatre nurse circulating	41,038	0.75	31	298.0	155	234.0	121	231.0	120
Portering staff	33,356	0.65	29	50.0	24	40.0	20	40.0	20
HCA	31,732	0.65	28	45.0	21	45.0	21	45.0	21

Total Staff cost					RATS		VATS		Open
					2396.4		1881.4		1856.8

Data sources:

Annual salary—HSE pay scales [16]

Operative time—literature review

**Table 3:** Consumable equipment costs by modality

Consumable	Units	Unit cost	Use RATS	Use VATS	Use Open
Maryland bipolar foreceps	Per case	170	1	0	0
Fenestrated bipolar foreceps	Per case	170	1	0	0
Permanent cautery hook (monopolar)	Per case	180	1	0	0
DaVinci Xi cadiere foreceps	Per case	90	1	0	0
Vessel Sealer	Per case	625	0.5	0	0
Staples RATS (staples + gun)	Per case	1917	1	0	0
Staples VATS/open (staples + gun)	Per case	1729	0	1	1
Sutures (total sutures)	Per case	15	1	1	1
Hemostatic consumables	Per case	32	1	1	1
Drapes	Per case	24	1	1	1
Scrub suit	Per case	9	2	3	3
Diathermy (consumables only)	Per case	185	1	1	1
Dressings	Per dressing	0	3	3	3
Chest drain and drainage system	Per case	72	1	1	1
Postop analgesia (paravertebral)	Per set	100	1	1	1
Blood transfusion	Per unit RCC	295	0.054	0.035	0.115

Total per-case consumable cost			RATS	VATS	Open
			3302.32	2196.66	2220.26

Data sources:

Consumable unit costs: outlined in table

Consumable usage: expert opinion (except blood transfusion rates—determined by literature review)

**Table 4:** Postoperative costs by modality, results presented for base case and the ‘Complications ± 20%’ scenarios

Resources	Resource unit cost	RATS use	RATS cost	VATS use	VATS cost	Open use	Open cost
Conversion to open	1000	0.081	81	0.126	126	0	0
Bed days recovery	500	1	500	1	500	1	500
Bed days critical care	1800	0.5	900	0.5	900	0.5	900
Bed days ward	856	4.8	4108.8	5.1	4365.6	6.2	5307.2
Physiotherapy	140	1	140	1	140	1	140
Lab tests	38	1	38	1	38	1	38
Chest X-rays	82	3	246	3	246	4	328
Minor complications	1000	0.38	380	0.418	418	0.457	457
Major complications	3000	0.059	177	0.078	234	0.123	369
Return to theatre	4000	0.032	128	0.032	128	0.039	156
Readmission	4500	0.067	301.5	0.082	369	0.068	306

Total postoperative cost			RATS		VATS		Open
Base case			7000.3		7464.6		8501.2
Complications + 20%			7112		7595		8666
Complications − 20%			6889		7334		8336

Data sources are presented in the table in the order ‘unit cost, resource use’

The cost components were divided into four categories: staff costs, consumables, postoperative costs and capital costs. Each of these categories is outlined in detail in the following sections.

### Data sources for systematic literature review

**Fig. 1:**
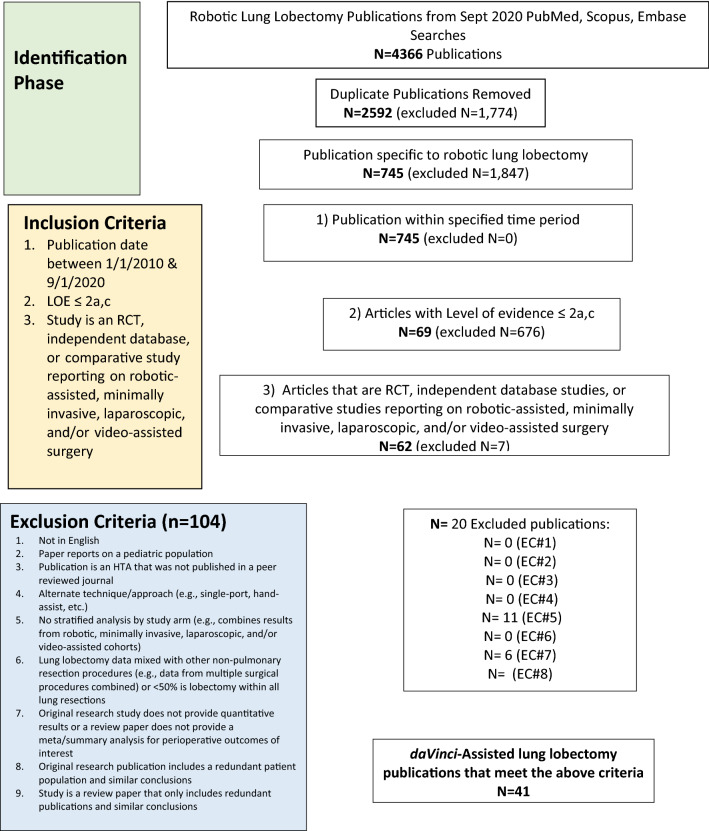
Robotic-assisted lung lobectomy flowchart (Search dates 1/1/2010–1/9/2020)

A systematic review was conducted in accordance with the PRISMA guidelines [14]. This search was updated based on the prior work of O’Sullivan and colleagues (2019) [9]. Searches were conducted using PubMed, Scopus and Embase databases to identify relevant publications for this clinical evaluation. The specific searches and search terms used were conducted as described in Table A1–A3. (Supplementary material: Appendix 1). All citations returned from the searches were exported into an EndNote library. The inclusion criteria were met if the publication pertained to robotic lobectomy surgery for lung cancer using the da Vinci Surgical System, published between January 1, 2010 and September 1, 2020, and was either a randomised controlled trial, meta-analysis, systematic review or database study with stratified analyses for robotic-assisted lobectomy, video-assisted lobectomy or open lobectomy. Exclusion criteria for the systematic review included; publications not in English language, health technology assessments that were not published in peer-reviewed journals, publications pertaining to a paediatric population, alternate surgical techniques such as single-port surgery or hand-assisted surgery, publications where stratified analyses by surgical approach were not provided, lobectomy procedures mixed with other thoracic procedures within a publication. Publications were further excluded if there were no quantitative data on perioperative outcomes, if original publications included redundant populations with similar conclusions or if review publications contained redundant publications and similar conclusions. A flowchart describing the included and excluded publications is provided (Fig. 1). Two reviewers independently extracted the clinical data from all relevant publications. Discrepancies were resolved prior to computations of weighted averages used as clinical inputs for the micro-costing models. Weighted averages and weighted standard deviations and 95% confidence intervals were computed using SAS version 9.4, (Carry NC, USA).

### Staff costs—time allocation

The ‘time allocation’ for each staff category was first determined by expert opinion. This accounted for the fact that different staff members were involved in different parts of the procedure, e.g. while the surgeon may be present for the entire ‘knife to skin’ time, they are not typically present for the entire preparation or post-operative time. ‘Time periods’ were then determined. A standard preparation time of 30 min for RATS, and 20 min for VATS and Open was assumed. Post-operative and ‘cleaning’ time periods, at 20 and 15 min, respectively, were assumed identical for each approach. The ‘knife to skin’ time period was derived from a systematic review of the literature.

The product of the staff-specific time allocations and the time periods were used to get the ‘time’ for each staff member per modality. For example, it was estimated that the surgeon is involved in half of the prep time (0.5), all of the ‘knife to skin time’ (1), half of the postop time (0.5) and none of the cleaning time (0) for a RATS lobectomy. Therefore, the ‘time’ that the surgeon is involved in a RATS lobectomy for is 0.5(30) + 1(247.6) + 0.5(20) + 0(15) = 272.6.

### Staff time and cost

Data for staff unit costs were derived from the Irish Department of Health Consolidated Salary Scales for 2018. The annual salary was divided by 45 working weeks per year and then by 39 working hours per week to give an hourly rate. A load factor was then applied as outlined in Table 2, to reflect the typical amount of patient contact time for each staff category. A personnel ‘hourly cost’ was then determined. This was then multiplied by the unique staff ‘time participation factor’, which aims to reflect the proportion of time each staff category is involved in the operation for (Table 1). Total/summative staff costs were then determined (Table 2).

Operative time and consequent staff costs were identified as an area in which it would be useful to analyse the effect of statistical uncertainty, given that operative time is a very surgeon specific, and to a lesser extent centre-specific metric, and where the systemic literature review provided measures of statistical uncertainty. As such a sensitivity analysis was performed for staff costs. The 95% confidence intervals for knife to skin time were obtained from the literature review. These were then applied to the model to determine the consequent variation in staff costs.

### Consumables

Resource use for consumables and equipment was largely determined by the expert opinion of a thoracic surgeon experienced in robotic, VATS and open lobectomy advising on the resource use in a typical cases for each approach (Table 4). The exception to this were blood transfusion rates, which were derived from a literature review. Unit costs were provided by Intuitive Surgical, Beacon Hospital Dublin, the UK National Institute for Health and Care Excellence (NICE), and the Irish Blood Transfusion Service (IBTS) [10]. All unit costs were on a ‘per case’ basis—e.g. ‘Staples RATS’ was the cost of the total staples and gun used in one case—this was determined by expert opinion. The consumable robotic instruments have a defined lifespan (number of cases), and thus the unit cost for these is the cost of each instrument divided by the assigned lifespan for that instrument.

### Post-op cost

Mean length of stay per approach was determined by literature review. It was assumed that 1 in 2 patients spent 1 day in ICU, and that this was the same across all approaches. This assumption was made due to the paucity of data in the existing literature regarding postoperative ICU length of stay after lobectomy by surgical approach. It was also recognised that whether a patient is managed in ICU post lobectomy is a very centre-specific question; in some centres these patients would be managed at ward or HDU level instead. A cost of €500 was attributed to time in ‘recovery’, or post-anaesthetic care, and this was assumed to be the same across all approaches. Physiotherapy time was evaluated using a single centre retrospective audit of practice over a 2-week period. Use of investigations, such as blood tests and chest X-rays, was estimated by the expert panel. Minor and major complication rates, as well as return to theatre and readmission rates were determined by literature review.

Costs for inpatient bed days at ward and ICU level were obtained from the Healthcare Pricing Office of the Health Services Executive [15]. The expert panel estimated the increased length of stay, at ICU and ward level, that major and minor complications following lobectomy were likely to produce, as well as the additional investigations. The costs for the additional length of stay and the additional investigations were then summated and used to represent the additional cost per patient that the complication was likely to produce. Costs for lab tests (Full Blood Count, Renal Profile, C-Reactive Protein) and chest X-rays were provided by Beacon Hospital, Dublin, and costs for physiotherapy time were derived from HSE salary scales [16].

A sensitivity analysis was applied to the cost of complications, to analyse the impact on the overall postop cost of increasing or decreasing the cost of complications by 20%. The results are presented in Table 4.

**Table 5:** Capital costs by modality

Cost:	RATS	VATS	Open
Purchase price	1,850,000	81,230	5000
Scrap value	150,000	0	0
Lifetime (years)	10	10	2
Maintenance cost (annual)	200,000	5686	0

Totals			
Equivalent annual capital cost	420,157.77	16,205.66	2689.03
Capital cost per procedure	622.45	24.87	3.98

### Capital costs

Capital and maintenance costs for robotic equipment were provided by Intuitive Surgical (Table 5). This included the capital cost of the unique sterilisation machine necessary for processing robotic instruments. Capital and maintenance costs for VATS equipment were provided by Irish Hospital Supplies Ltd. Cost for an open instrument set is included for all three modalities, as all minimally invasive procedures (both RATS and VATS) need the capability to convert to open as necessary.

Several assumptions were made regarding the capital cost calculations. The model assumed that three procedures were performed per day, 5 days per week, 45 weeks of the year. A discount rate of 5% was applied.

To further illustrate the contribution made by the ‘cost components’ to the overall cost difference, a threshold analysis was performed. This was felt to be useful in that it would demonstrate clearly the proportion by which robotic cost components would have to be reduced to achieve cost equality with VATS. The overall cost difference between the RATS and VATS approaches was €1754. This was subtracted from each of the staff cost, consumable and postoperative cost drivers. Capital cost was not included in the threshold analysis as the difference between RATS and VATS was less than the overall cost difference. The resulting figure was then expressed as a percentage of the RATS cost component.

Statistical analysis was not applied to the model as a whole. This analysis used a deterministic costing model with no individual patient-related observations available for analysis. Furthermore, data were drawn from several different sources, including from expert opinion. Due to the nature of the analysis as a deterministic cost model, and to the heterogeneity of data sources, an overall statistical analysis was not appropriate. For sections of the model, weighted averages of estimates were derived from the updated systematic literature review. Statistical analysis on these was performed using SAS version 9.4 (North Carolina, USA).

## Results

**Fig. 2:**
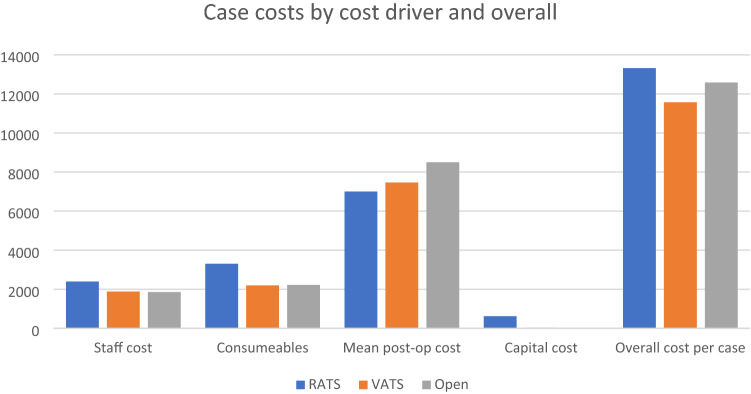
Case costs by cost driver and overall

Total and aggregate costs of each of the four cost components were summated for each approach as outlined in Fig. 2. Total cost per case for the RATS approach was €13,321 for the VATS approach was €11,567, and for the Open approach was €12,582.

The results of the sensitivity analysis for the cost of complications are presented in Table 4 (‘complications ± 20% scenarios). The effect of increasing the cost of complications by 20% did not cause a significant increase in overall postoperative costs (1.6% increase), and even less of an increase in total cost (0.8% increase).

The results of the sensitivity analysis for staff costs are outlined in Table 6. These figures serve to illustrate the statistical uncertainty regarding knife to skin time, which is likely to be a cost driver of interest to service providers given the variation between surgeons, and how operative time may be affected by the learning curve, with procedures becoming shorter as operators become more proficient.

**Table 6:** Sensitivity analysis for staff costs

	RATS	VATS	Open
Overall cost	2396	1881	1856
95% confidence interval	1634–3147	1283–2495	1023–2693
Median	2397	1878	1864

The results of the threshold analysis for overall costs are outlined in Table 7. To achieve cost equality between the approaches, RATS staff costs would need to be reduced by 73%, consumables by 53% or postoperative costs by 25%. Of course in reality cost equality would likely be achieved by reducing all three cost components by varying amounts.

**Table 7:** Threshold analysis

	RATS	VATS	RATS-1754	Need to reduce by to achieve equity (%)
Staff cost	2396	1881	642	73
Consumables	3302	2196	1548	53
Postoperative cost	7000	7464	5246	25
Capital cost	622	24		
Overall cost	13,321	11,567		
Difference to achieve equity	1754			

## Discussion

In dividing the cost drivers/resources in our model into four components, we set out to evaluate specifically what drives the increased cost of RATS lobectomy. It is important to note that the scope of our analysis was not to evaluate the relationship between cost and patient outcomes. We analysed the cost using a deterministic cost model and did not conduct a cost-effectiveness analysis.

Our analysis suggests that the higher cost of robotic surgery is driven more by the increased cost of consumable equipment than by anything else, with robotic consumables being €1106 more expensive per case than VATS consumables. Increased staff costs, driven by the significantly longer ‘knife to skin’ time with RATS vs VATS, also contribute €515 to the difference between the two approaches.

By contrast, the difference in per-case capital costs between RATS and VATS is a mere €598, or 34% of the total cost difference between the approaches. This would suggest that, while efficient use of the robotic equipment is important, even if a programme was to double the use of their robotic equipment (i.e., perform 6 sessions per day instead of 3, use the equipment at night, etc.), the impact on the per-case cost difference would not be significant, reducing it by just 16%. It would also suggest that the difference in the per-case cost difference should the capital costs be removed entirely (i.e., if the robotic equipment is donated by a charitable entity), would not change the relative cost variation substantially.

Moreover, what quickly becomes apparent on evaluation of the results is that the significantly more expensive robotic consumables contribute substantially to the overall cost difference, accounting for 63% of the overall cost difference. Most of this cost difference is accounted for by the cost of proprietary daVinci consumable equipment—instruments, staplers, etc. It is worth noting that the instrument costs are all calculated on a ‘per use’ basis—i.e., that the instrument may be used a defined number of times only before being retired. The use limitations are set by Intuitive Surgical, manufacturer of the daVinci Surgical System.

The high cost of consumable equipment, and in particular of staplers, is not a unique problem to robotic surgery. Indeed, in several cost analyses comparing VATS to open lobectomy, disposable costs, and in particular the increased utilisation of endo staplers, are highlighted as one of the most significant factors in the increased cost associated with VATS lobectomy [17–19]. In this analysis, we have explicitly addressed the challenges of allocating fixed cost of equipment as average cost per patient. These challenges are present in all cost analyses related to both clinical trials and managerial explorations. In contrast to many analysts who disregard equipment costs, we have been explicit and transparent in our assessment of the assumed equipment cost.

Micro-costing has distinct advantages over a ‘top–down’ costing approach (for example, the use of Diagnosis Related Groups (DRGs) to allocate funding in Irish Healthcare). As Potter et al. (2020) point out, micro-costing allows for comparison of different approaches to the same procedure (as is the case with this study). This makes micro-costing particularly useful in the evaluation of surgical innovation, where it is often a small change in a procedure, as opposed to a comparison to an entirely separate procedure, that one wants to evaluate. It also allows investigators to tailor studies and focus on identifying and costing key areas where there is a significant cost difference (for example in this study with consumable equipment costs) [20]. A major strength of this approach is that it allows for the testing of different assumptions to ascertain their impact on total treatment cost.

The consequent focus on incremental cost difference allows for the study results to be relevant to a broader number of jurisdictions, as the overall cost of the intervention or procedure (which varies significantly depending on local unit costs) is less relevant. As such the findings of this study are likely to be generalisable outside of the Irish context, certainly in the United Kingdom and the European Union. The overall patient pathway for lobectomy is similar in these jurisdictions, and thus the model outlined above could be applied (with some refinement of unit cost inputs to reflect local salary scales etc.).

There are several limitations of this analysis. The preoperative clinical condition of the patient is not accounted for, and indeed in other specialties, such as gynaecology, it has been suggested that the higher cost of a robotic approach is influenced by the fact that patients undergoing robotic surgery tend to be more comorbid than patients undergoing laparoscopic surgery, and not just by the surgical approach alone [21]. Furthermore longer-term clinical and economic outcomes were not evaluated. Evidence from other specialties would suggest that the robotic approach has advantages in terms of decreased long-term opiate use, and faster return to work [22–24]. Factors such as these were not considered in this analysis.

In conclusion, this study presents a detailed analysis of the hospital cost for lobectomy, evaluated for the three primary surgical approaches. We offer this analysis as a useful tool for surgeons, hospital management, and service commissioning agencies to accurately and comprehensively determine where cost savings can be applied in their programme, to maximise the cost efficiency of a robotic lobectomy.

## Supplementary Information

Below is the link to the electronic supplementary material.

Supplementary file1 (DOCX 15 KB)

## Data Availability

All relevant data are within the manuscript and its Supporting Information files.
