# Supplementary material for: Is robotic lobectomy cheaper? A micro-cost analysis
Source: J Robot Surg. 2022 Feb 28;16(6):1441–50. doi: 10.1007/s11701-022-01377-x (PMC9606087; doi:10.1007/s11701-022-01377-x)
Supplement: Supplementary file 1 — Supplementary file1 (DOCX 15 KB) [file 11701_2022_1377_MOESM1_ESM.docx]

**Supplementary Material:**

**Appendix 1: Pubmed, Scopus and Embase Lobectomy Literature Searches (1/1/2010 to 09/01/2020)**

| **A1** | | **Pubmed Search Terms** |
| --- | --- | --- |
|  | | (robotic[All Fields] OR robot*[All Fields] OR robot assist[All Fields] OR robotically assisted[All Fields] OR robot-assist[All Fields] OR "da vinci"[All Fields] OR davinci[All Fields] OR "intuitive surgical"[All Fields] OR (robot*[All Fields] AND surgery[All Fields])) AND (((“lung cancer” OR “lung resection” OR pneumonectomy OR segmentectomy OR “pulmonary resection” OR lobectomy) OR ((lung OR pulmonary) AND (lobectomy OR resection OR pneumonectomy OR segmentectomy OR wedge OR thoracic OR thoracoscopic)) OR (lobectomy))) AND (("2010/01/01"[Date - Publication] : "2020/09/01"[Date - Publication]))) AND (("2019/10/31"[Date - Entry] : "2020/09/01"[Date - Entry])))) AND (English[Language]) |
|  | |  |
|  |  | |
|  |  | |
| **A2** | **Scopus Search Terms** | |
|  | ( ( ( TITLE-ABS-KEY ( da*vinci ) OR ( robotic AND surgery ) OR ( "intuitive surgical" ) OR ( robotic AND assist* ) OR ( robot*surgery ) OR ( robotic-assist* ) ) OR ( TITLE-ABS-KEY ( ( da*vinci ) OR ( intuitive W/3 surgical ) OR ( robot* W/2 assist* ) OR ( robot*assist* ) OR ( robot* W/8 ( surgery OR surgeries OR surgical* OR bypass OR graft OR sentinel OR *ectomy OR *otomy OR *plasty OR *opexy OR cancer OR tumor OR neoplasm* OR excis* OR anastomosis OR benign OR malignanc* ) ) OR endowrist OR 'dv-trainer' ) OR MANUFACTURER ( intuitive ) OR TRADENAME ( 'da AND vinci' OR davinci ) OR AFFIL ( intuitive OR intusurg ) OR REFTITLE ( ( da*vinci ) OR ( intuitive W/3 surgical ) OR ( robot*assist* ) OR ( ( robot* ) W/8 ( surgery OR surgeries OR surgical OR cancer OR bypass OR graft OR sentinel OR *ectomy OR *otomy OR *plasty OR *opexy OR tumor OR cancer OR neoplasm* ) ) OR endowrist OR 'dv-trainer' ) OR ( EXACTSRCTITLE ( 'health AND technology AND assessment' ) AND TITLE-ABS-KEY ( surgery OR surgeries OR surgical ) ) ) ) AND ( ( TITLE-ABS-KEY ( ( ( lung OR pulmonary ) W/15 ( resect* OR surgery OR surgical* OR surgeries OR segmentectom* OR wedge OR lobectom* OR thoracic OR thoracoscop* ) ) OR pneumonectom* OR lobectom* ) ) OR ( ( REF ( ( robot OR robotic OR robotically ) AND ( ( ( lung OR pulmonary ) W/3 ( resect* OR surgery OR surgical* OR surgeries OR segmentectom* OR wedge OR lobectom* OR thoracic OR thoracoscop* ) ) OR pneumonectom* OR lobectom* ) ) AND NOT TITLE ( thyroid* ) ) ) ) ) OR ( ( TITLE-ABS-KEY ( ( robot OR robotic OR robotically ) W/8 ( lobectom* OR thoracic OR thoracoscop* ) ) AND ALL ( ( lung OR pulmonary ) ) ) ) AND ( ( PUBYEAR > 2019 AND PUBYEAR < 2021 ) OR ( ( PUBDATETXT ( november 2019 ) ) OR ( PUBDATETXT ( december 2019 ) ) ) OR ORIG-LOAD-DATE > 20191031 ) AND ( EXCLUDE ( SRCTYPE , "b" ) OR EXCLUDE ( SRCTYPE , "k" ) ) AND ( LIMIT-TO ( LANGUAGE , "English" ) ) | |

| **A3** | **Embase Search Terms** |
| --- | --- |
|  | ((('da vinci':de,nc,lnk,cl,ab,ti OR 'davinci':de,nc,lnk,cl,ab,ti OR 'intuitive surgical' OR 'endowrist'/exp OR endowrist OR ((robot* NEAR/2 surg*):de,nc,lnk,cl,ab,ti) OR (robot* NEXT/1 assist*)) NOT (arthropl* OR 'gait'/exp OR gait) AND ((((lung OR pulmonary) NEAR/15 (resect* OR surgery OR surgical* OR surgeries OR segmentectom* OR wedge OR lobectom* OR thoracic OR thoracoscop*)):ti,ab,kw) OR pneumonectom*:ti,ab,kw OR lobectom*:ti,ab,kw OR ((resect*:ti,ab,kw OR surgery:ti,ab,kw OR surgical*:ti,ab,kw OR surgeries:ti,ab,kw OR segmentectom*:ti,ab,kw OR wedge:ti,ab,kw OR lobectom*:ti,ab,kw OR thoracic:ti,ab,kw OR thoracoscop*:ti,ab,kw OR pneumonectom*:ti,ab,kw) AND 'lung cancer'/exp)) OR ((((robot OR robotic OR robotically) NEAR/8 (lobectom* OR thoracic OR thoracoscop*)):ti,ab,kw) AND (lung OR pulmonary))) AND [2019-2020]/py OR ((('da vinci':de,nc,lnk,cl,ab,ti OR 'davinci':de,nc,lnk,cl,ab,ti OR 'intuitive surgical' OR 'endowrist'/exp OR endowrist OR ((robot* NEAR/2 surg*):de,nc,lnk,cl,ab,ti) OR (robot* NEXT/1 assist*)) NOT (arthropl* OR 'gait'/exp OR gait) AND ((((lung OR pulmonary) NEAR/15 (resect* OR surgery OR surgical* OR surgeries OR segmentectom* OR wedge OR lobectom* OR thoracic OR thoracoscop*)):ti,ab,kw) OR pneumonectom*:ti,ab,kw OR lobectom*:ti,ab,kw OR ((resect*:ti,ab,kw OR surgery:ti,ab,kw OR surgical*:ti,ab,kw OR surgeries:ti,ab,kw OR segmentectom*:ti,ab,kw OR wedge:ti,ab,kw OR lobectom*:ti,ab,kw OR thoracic:ti,ab,kw OR thoracoscop*:ti,ab,kw OR pneumonectom*:ti,ab,kw) AND 'lung cancer'/exp)) OR ((((robot OR robotic OR robotically) NEAR/8 (lobectom* OR thoracic OR thoracoscop*)):ti,ab,kw) AND (lung OR pulmonary))) AND [31-10-2019]/sd NOT [2-9-2020]/sd AND [2010-2020]/py)) AND [english]/lim NOT [conference abstract]/lim |
